# The Optimal Dosage of Fermented Herbal Extract on Growth and Feed Efficiency of Nile Tilapia (*Oreochromis niloticus*)

**DOI:** 10.21315/tlsr2023.34.2.3

**Published:** 2023-07-21

**Authors:** Yushinta Fujaya, Andi Aliah Hidayani, Dwi Kesuma Sari, Siti Aslamyah, Nita Rukminasari, Abdul Muthalib, Stevie Cristianto, Emilia Defista, Hanafiah Fazhan, Khor Waiho

**Affiliations:** 1 Fisheries Department, Faculty of Marine Science and Fisheries, Hasanuddin University, Jl. Perintis Kemerdekaan Km. 10, Makassar, South Sulawesi, Indonesia; 2 Study Program of Veterinary Medicine, Faculty of Medicine, Hasanuddin University, Jl. Perintis Kemerdekaan Km. 10, Makassar, South Sulawesi, Indonesia; 3 Higher Institution Centre of Excellence (HICoE), Institute of Tropical Aquaculture and Fisheries, Universiti Malaysia Terengganu, Kuala Nerus, 21300 Kuala Terengganu, Terengganu, Malaysia; 4 Centre for Chemical Biology, Universiti Sains Malaysia, Sains@USM, Blok B No. 10, Persiaran Bukit Jambul, 11900 Bayan Lepas, Pulau Pinang, Malaysia

**Keywords:** Feed Efficiency, Fermented Herbal Extracts, Intestinal Changes, Nutrient Retention, Tilapia, Kecekapan Makanan, Ekstrak Herba yang Ditapai, Perubahan Usus, Pengekalan Nutrien, Tilapia

## Abstract

Owing to their availability, cost effectiveness and environmental-friendly nature, plant extracts are promising additives for fish farming. This study aims to determine the optimal dosage of fermented herbal extract (FHE)—composed of *Morus alba* (33.3%), *Curcuma xanthorrhiza* (33.3%), and *Boesenbergia rotunda* (33.3%)—for growth enhancement and feed utilisation efficiency of *Oreochromis niloticus* fingerlings. Fermentation was conducted using probiotics *Lactobacillus casei* (Yakult®, Tokyo, Japan) and *Saccharomyces cereviceae* (commercial baker’s yeast). The FHE was high in flavonoid and alkaloid, vitamin C, potassium, natrium, lipase and protease. Four doses of FHE treatments, namely treatment A (0 mg/kg of feed); treatment B (100 mg/kg of feed); treatment C (300 mg/kg of feed); treatment D (500 mg/kg of feed) were compared. After subjected to 35 days of culture, tilapias subjected to FHE-coated feed exhibited better weight gain (WG), specific growth rate (SGR), and feed efficiency (FE) compared to control. The best dosage that gave the highest growth and feed efficiency was treatment C (300 mg/kg of feed). Furthermore, the feed efficiencies of FHE-incorporated treatments were positively influenced by the increased in length and density of intestinal villi, number of goblet cells, lymphocytes, as well as nutrient retention to support growth. The results of this study indicate that FHE is a promising functional feed additive to stimulate growth and improve feed efficiency in tilapia farming.

## Abstrak

Disebabkan ketersediaan, keberkesanan kos dan sifat mesra alamnya, ekstrak tumbuhan ialah bahan tambahan yang mempunyai harapan cerah dalam penternakan ikan. Kajian ini bertujuan untuk menentukan dos optimum ekstrak herba yang ditapai (FHE)— yang terdiri daripada *Morus alba* (33.3%) *Curcuma xanthorrhiza* (33.3%) dan *Boesenbergia rotunda* (33.3%)—untuk peningkatan pertumbuhan dan kecekapan penggunaan makanan *Oreochromis niloticus* ikan kecil. Penapaian dijalankan menggunakan probiotik *Lactobacillus casei* (Yakult®, Tokyo, Jepun) dan *Saccharomyces cereviceae* (yis pembuat roti komersial). FHE tinggi dalam flavonoid dan alkaloid, vitamin C, kalium, natrium, lipase dan protease. Empat dos rawatan FHE, iaitu rawatan A (0 mg/kg makanan); rawatan B (100 mg/kg makanan); rawatan C (300 mg/kg makanan); rawatan D (500 mg/kg makanan) dibandingkan. Selepas menjalani kultur selama 35 hari, tilapia yang diberi makanan bersalut FHE menunjukkan peningkatan berat (WG), kadar pertumbuhan spesifik (SGR) dan kecekapan makanan (FE) yang lebih baik berbanding tilapia kawalan. Dos terbaik yang memberikan pertumbuhan tertinggi dan kecekapan makanan adalah rawatan C (300 mg/kg makanan). Tambahan pula, kecekapan suapan rawatan yang diperbadankan FHE dipengaruhi secara positif oleh peningkatan panjang dan ketumpatan vilus usus, bilangan sel goblet, limfosit, serta pengekalan nutrien untuk menyokong pertumbuhan. Hasil kajian ini menunjukkan bahawa FHE ialah bahan tambahan makanan berfungsi yang menjanjikan untuk merangsang pertumbuhan dan meningkatkan kecekapan makanan dalam penternakan tilapia.

**Highlights**

- Fermented herbal extract (FHE) composed of *Morus alba* (33.3%), *Curcuma xanthorrhiza* (33.3%) and *Boesenbergia rotunda* (33.3%) was high in flavonoid and alkaloid, vitamin C, potassium, natrium, lipase and protease.
- FHE-coated feed enhanced weight gain, growth and feed efficiency.
- Treatment C (300 mg/kg of feed) gave the highest growth and feed efficiency.

## INTRODUCTION

Aquaculture plays an important role in sustaining the livelihood, employment and local economic development of coastal communities in developing countries. It accounts for 46% of total fish production and 52% for human consumption (FAO 2020a). However, with the rapid intensification of the aquaculture sector, cases of diseases and crop failures increased tremendously, causing serious losses for farmers (Waiho *et al*. 2021a). The use of antibiotics and other synthetic compounds for treatment and stimulating growth play a key role in the increasing prevalence of diseases caused by resistant bacteria (Suphoronski *et al*. 2019). The use of chemical drugs also has several negative impacts on the environment and humans (Boyd & McNevin 2014). Therefore, in recent decades, the need for sustainable cultivation of aquatic organisms by avoiding the use of antibiotics and synthetic chemical compounds as growth promoters have become increasingly necessary (FAO 2020b).

Nile tilapia (*Oreochromis niloticus*) is one of the most important aquaculture species of the 21st century and traded globally in more than 125 countries (Tran *et al*. 2021). Its production in 2019 was estimated at 6.5 million metric tons, and a growth of 4% compared to 2018. Owing to its fast growth rate and sturdy nature, tilapias are being regarded as essential food fish for most developing countries and play roles in global food and nutrition security, contributing significantly towards the United Nation’s Sustainable Development Goals (SDGs) of “No Poverty” and “Zero Hunger” (Lynch *et al*. 2020). However, farmed tilapias are susceptible to diseases that would often lead to mass mortality, especially when cultured in intensive systems (Chitmanat *et al*. 2016). In 2019, the loss of an approximately 300,000 metric tons of tilapias were disease-related (FAO 2021). Being the second largest tilapia producer in the world, Indonesia experienced a decline in production of 7.45% in 2019 compared to the two-decade highest peak production in 2017 (1,280,127 t) (FAO 2020a).

To ensure sustainability and remain eco-friendly, the use of alternatives to antibiotics and synthetic growth promoters are required in the aquaculture sector. One alternative is the use of natural herbs that have medicinal value. Natural herbs are known to possess active compounds that are useful in improving the performance of aquaculture production, including as natural or non-antibiotic growth promoters. There are currently over 60 species of medicinal plants with strong pharmacological and therapeutic features being used in the aquaculture industry (Latif *et al*. 2021), and many more are showing great potential for future incorporation into fish and shellfish farming (Moh *et al*. 2021; Wu *et al*. 2021). In addition to being biodegradable, cheap, readily available, and environmentally friendly, plants are promising alternatives as synthetic hormones that could be applied in the aquaculture industry (e.g., phytoecdysteroid in plants can be used to replace ecdysteroid to induce moulting in crustaceans) (Waiho *et al*. 2021b). Plant extracts are also being used with probiotics to produce synbiotics that could enhance fish growth and improve immune response. For example, extracts of hairy eggplant *Solanum ferox*, when mixed with probiotic *Lactobacillus casei*, improved both growth indices and immunity in catfish *Clarias gariepinus* (Hardi *et al*. 2022).

Previous studies have reported promising effects of herbal extracts on the growth of tilapia, *O. niloticus*. Palipoch *et al*. (2011) reported that *Thunbergia laurifolia* leaves can reduce the toxicity of lead nitrate (Pb(NO_3_)_2_) and increase the growth performance of *O. niloticus*. The use of ethanol extracts of several common Indonesian plant species (i.e., *Boesenbergia pandurata*, *Solanum ferox*, *Zingiber zerumbet*) improved the nonspecific immunity and enhanced protection against *Aeromonas hydrophila* and *Pseudomonas* sp. infections in *O. niloticus* (Hardi *et al*. 2017). The growth and immunity of *O. niloticus* was also significantly enhanced when subjected to the crude extracts of *Camellia sinensis*, *Aloe vera*, *Cinnamomum camphora*, *Euphorbia hirta*, *Azadirachta indica* and *Carica papaya* (Abdel-Tawwab *et al*. 2010; Gabriel *et al*. 2015; Kareem *et al*. 2016; for review on the herbal extracts used in tilapia culture, see Gabriel 2019). Apart from growth and immunity, herbal extracts such as *A. vera* and ginseng are known to improve the hematological parameters of *O. niloticus* (Goda 2008; Gabriel *et al*. 2015). Most studies further showed that herbal extracts exert their beneficial effect onto fish in a dose-dependent manner, and the positive impact is often directly correlated until the optimum inclusion level (Gabriel 2019).

Indonesia is one of the countries with high diversity of herbal plants. Since ancient times, Indonesians have used herbs to treat diseases, increase appetite and boost immunity (Elfahmi *et al*. 2014). Some very popular medicinal herbs include *temu lawak* (*Curcuma xanthorrhiza*) and *temu kunci* (*Boesenbergia rotunda*); the former is a medicinal plant that contains curcumin and xanthorrhizol (Oon *et al*. 2015) and known to exert diverse physiological functions, including as appetite stimulant, hepatoprotection, antimicrobial, anti-inflammatory, analgesic, antipyretic, chloretic, and others, while the latter is used as “jamu” to improve intestinal health (Eng-Chong *et al*. 2012). Mulberry *Morus alba* is less popular for medicinal purposes, but it is the main feed for silkworm farms. *M. alba* has abundant phytochemicals and bioactive compounds that are potential pharmacological agents against diseases and growth enhancers (Chen *et al*. 2021; Das *et al*. 2021). Recently, mulberry leaf extract has been successfully trialled as a growth and moulting stimulant of mangrove crabs (Fujaya *et al*. 2018).

Owing to the high potential of herbal plant extracts in growth enhancement, this study aims to examine the effect of a combination of local herbal extracts (*M. alba, C. xanthorrhiza* and *B. rotunda*) as a feed additive for the culture of *O. niloticus* fingerlings. Specifically, this study determines the optimal dose of the herbal extract combination fermented with *Lactobacillus* sp. and *Saccharomyces cerevicae*, also known as fermented herbal extract (FHE). It was expected that the active ingredients of the FHE would support and promote the growth and feed efficiency of tilapia. The FHE effect on the appearance of intestinal histology and nutrient retention was also discussed to provide a deeper understanding on the impact of herbal extracts on fish feed efficiency and overall growth.

## MATERIALS AND METHOD

### Fish Culture

This study was conducted at the Fish Hatchery Technology Laboratory, Faculty of Marine and Fisheries Sciences, Hasanuddin University, Indonesia. Approximately 1,000 tilapia fingerlings (average weight: 13.25 ± 1.10 g; unsexed) were obtained from the Takalar Regency and acclimatised in a stocking tank with a volume of 250 L for 14 days. After acclimatisation, the fish were randomly distributed to different treatments, with each treatment replicate was made up of 105 fishes distributed equally into three conical tanks filled with 200 L of water in each tank. All tanks were housed in the hatchery exposed to similar photoperiod (12 h light: 12 h dark). Water was treated with chlorine and neutralised with sodium thiosulfate before use. Each flask was equipped with continuous aeration and natural photoperiod was applied. Half of the water volume was exchanged every week to maintain water quality. All experimental procedures comply with the ARRIVE (Animal Research: Reporting of In Vivo Experiments) guidelines and carried out in accordance with the UK legislation under the Animals (Scientific Procedures) Act 1986 Amendment Regulations (SI 2012/3039) and associated guidelines, EU Directive 2010/63/EU for animal experiments. This study also followed the guidelines of the Committee of Animal Welfare and Research Ethics, Hasanuddin University, Indonesia and approved by the Faculty of Marine Science and Fisheries, Hasanuddin University.

### Feed Formulation

The base feed used in this study was a commercial pellet (ALL FEED; manufacturer: PT Central Proteina Prima Tbk., Indonesia). Proximate analysis was conducted following the standard AOAC protocol 2005 to determine the moisture, crude protein, crude lipid, ash, crude fibre and nitrogen free extract (Table 1) (Wu *et al*. 2021). A Completely Randomised Design (CRD) was applied. Four treatments were used in this study, in terms of FHE-coated feed, namely: (A) 0 mg/kg of feed (control); (B) 100 mg/kg; (C) 300 mg/kg and (D) 500 mg/kg of feed. All treatments were conducted in triplicates. FHE was made up of three herbal extracts, i.e., mulberry leaf extract (*Morus alba*), *temulawak* (*Curcuma xanthorrhiza)*, and *temu kunci* (*Boesenbergia* rotunda), at equal concentrations, and fermented with probiotics (*Lactobacillus casei* and *Saccharomyces cereviceae*). The probiotic strain of *L. casei* was obtained from Yakult ® (Tokyo, Japan for human consumption) whereas commercial baker’s yeast, *S. cerevisae* in the form of instant dried yeast was used. In brief, approximately 1 mL of diluted *S. cerevisae*, 1 mL of *L. casei* (4 × 10^6^ cfu/mL) and 1 mL of pre-treated (boiled) molasses were added into 1 L of distilled water containing 500 mg of herbal extracts (Stock FHE; fermentation time: 30 days; fermentation condition: 29°C–32°C). The dilution of stock FHE for each treatment was as follows: treatment A (100 mL of distilled water); treatment B (20 mL FHE stock + 80 mL distilled water); treatment C (60 mL FHE Stock + 40 mL distilled water); treatment D (100 mL FHE stock; without distilled water).

The biochemical and phytochemical properties of the concocted FHE was determined. Sitosterol and curcumin were analysed using thin-layer chromatography whereas alkaloid, saponin and flavonoid were determined using ultraviolet-visible (UV-Vis) spectrophotometry, both performed by the Integrated Research and Testing Laboratory of Gadjah Mada University, Indonesia. Vitamins and minerals were determined using spectrophotometry and atomic absorption spectroscopy, respectively, by Makassar Health Laboratory Centre, Indonesia. Protease assay, amylase assay and lipase assay were carried out by the Biochemical Laboratory of FMIPA-UNHAS, Indonesia. In brief, FHE contained sitosterol, alkaloids, saponin, flavonoid, curcumin, and several vitamins and minerals (Table 2). The base feed was coated with FHE according to the concentrations of the designed treatments by the spraying method (Siddik *et al*. 2022). After coating, the FHE-coated feed was air-dried and packaged according to the amount of daily feeding (5% body weight). The feeding was scheduled twice daily, which is morning and evening (0700–0800 and 1700–1800). Uneaten feed was retrieved an hour after feeding and weighed for the calculation of feed consumption. Fish rearing was carried out for 35 days.

### Growth, Feed Efficiency, Intestinal Histology and Nutrient Retention

The measured parameters include growth, feed efficiency and survival rate. Fish were anesthetised using clove power (200 ppm) and culled immediately using a percussive blow to the head (Anders *et al*. 2019). Intestinal histology analysis was carried out to obtain gut performance, villi length, the distance between villi and goblets cells and lymphocytes count. In addition, water quality parameters namely temperature, dissolved oxygen and ammonia content were measured using handheld thermometer, the Winkler Titration Method, and spectrophotometry, respectively as supporting parameters.

Growth parameters consisted of: weight gain (WG) as proxy for absolute growth, average daily growth (ADG), and specific growth rate (SGR). WG was the difference between the average final and the initial weight, while the ADG was WG divided by the number of rearing days. SGR was obtained by the formula:


$$\text{SGR}=\text{Ln WF}-\frac{\text{LN W}0}{t}\times 100$$

where W0 is the initial weight (g), WF is the final weight (g), and *t* is the cultivation period (day). Feed consumption (FC) was by the difference in the amount of feed given to the amount remaining based on dry weight. Feed efficiency (FE) was calculated with the formula:


$$\text{FE}=(\text{WF}+\text{D})-\frac{\text{W}0}{\text{FC}}\times 100$$

where WF is the final weight (g), W0 is initial weight (g), D is the weight of fish that died during rearing (g) and FC is the amount of feed consumed (g). The survival rate (SR) is the percentage of fish that lived at the end of the study.

Intestinal histology preparations were made and segments of approximately 1.5 cm were taken from the small intestine, gently washed with 0.9% NaCl to remove contents, and fixed in fresh of 4% formaldehyde for 48 h. All the samples were dehydrated, cleared and embedded in paraffin. Serial sections were cut at 4 μm and placed on glass slides. For all assays, sections were deparaffinised in xylene and rehydrated in a graded alcohol series. Furthermore, observations and images were taken using an advanced Opti lab microscope camera. From the image results, the observed cells which are the target of the study were scanned. The parameters observed include the length of villi, distance between villi, goblet cell and lymphocyte cell count.

Nutrient retention analysis was also carried out to help study the effect of herbal extracts on growth and feed efficiency. The nutrient retention analysed was protein, fat and energy according to the equations:


$$\begin{array}{l} \text{Protein Retention }(\text{PR})(\%)=\frac{\left(\left(\text{W}2\times \text{P}2)-(\text{W}1\times \text{P}2\right)\right)}{\text{Protein intake}}\times 100;\\ \text{Lipid Retention }(\text{LR})(\%)=\frac{\left(\left(\text{W}2\times \text{L}2)-(\text{W}1\times \text{L}1\right)\right)}{\text{Lipid intake}}\times 100;\\ \text{Energy Retention }(\text{ER})(\%)=\frac{\left(\left(\text{W}2\times \text{E}2)-(\text{W}1\times \text{E}1\right)\right)}{\text{Energy intake}}\times 100 \end{array}$$

where P1 and P2 are protein content in the initial and final body of fish, L1 and L2 are lipid content in initial and final body, E1 and E2 are energy content in initial and final body, and W1 and W2 are initial and final weight of fish. Raw measurement data can be found in Appendix.

### Data Analysis

The effect of treatment on the observed parameters was analysed by Analysis of Variance (ANOVA) and continued with the Least Significant Difference (LSD) test. Normality and homogeneity of variance were checked using Shapiro-Wilk test and Levene’s test, respectively. The analytical tool used was computer software packages, namely Microsoft Excel and IBM SPSS Statistics 20.0. Significant level was fixed at *P* = 0.05. In addition, the optimal doses were determined using a polynomial model trendline of Microsoft Excel.

## RESULTS

The measured water qualities (i.e., DO, temperature and ammonia) were in the optimal range and fluctuates minimally within the 35-day experimental period, with DO at 27.8 mg/L–30.0 mg/L, temperature at 29.2ºC–30.1ºC, and ammonia at 0.01 ppm–0.06 ppm. Tilapias exhibited higher WG and SGR when fed with feed coated with 100 mg/kg and 300 mg/kg of FHE (Table 3). Further, the inclusion of FHE on feed had negligible impact on the survival of tilapias. In average, the daily growth was not significant among treatments (*P* > 0.05). Similarly, tilapias fed with feed coated with 100 mg/kg and 300 mg/kg of FHE showed significantly better feed efficiencies, although the daily consumption of feed remained the same across treatments (Table 3).

**Figure 1:**
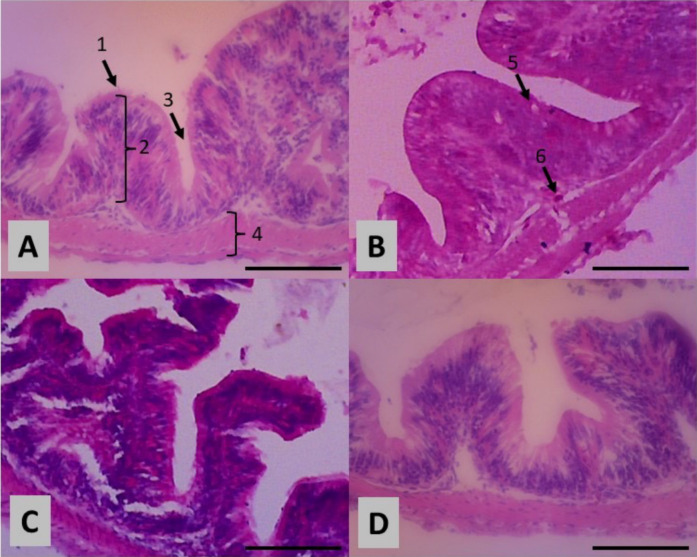
The intestinal histology of tilapia (*Oreochromis niloticus*) in treatment (A) Control, (B) 100 mg/kg of feed, (C) 300 mg/kg of feed, and (D) 500 mg/kg of feed. Staining method: Hematoxylin Eosin; Magnification: 40 × 10; Bar: 100 μm. (1) Intestinal villi of tilapia; (2) Length of villi; (3) Distance between villi; (4) Thickness of muscles; (5) Lymphocyte cells and (6) Goblet cells.

In general, the inclusion of FHE in the feed of tilapias caused significant intestinal changes. The length of intestinal villi was highest in treatment C (300 mg/kg of feed), followed by treatment B (100 mg/kg of feed), and lastly control (0 mg/kg of feed) and treatment D (500 mg/kg of feed) showed no difference between them (Table 3; Fig. 1). As the villi length increases, the distance between villi decreases in almost similar manner. Goblet cells were most abundant in treatment B, followed by treatment C, whereas control and treatment D had the lowest goblet cell count. Tilapias fed with FHE-treated feed had significantly higher lymphocyte cell counts per villi, with treatment B being the highest (Table 3).

**Figure 2:**
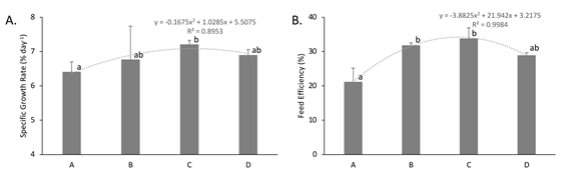
(A) The specific growth rate (SGR) and (B) feed efficiency (FE) of the four treatments of *Orechromis niloticus* after 35 days. Treatment A: 0 mg/kg of feed; treatment B: 100 mg/kg of feed; treatment C: 300 mg/kg of feed and treatment D: 500 mg/kg of feed. Error bars indicate standard errors. Superscript alphabets indicate significant difference between treatments (*P* < 0.05).

The polynomial growth model of fish based on the dose treatment of FHE was visualised in Fig. 2. The figure clearly showed that the dose of herbal extract affected growth (R^2^ = 0.9044; Fig. 2A) and feed efficiency (R^2^ = 0.9984; Fig. 2B), with treatment C (300 mg/kg of feed) being the optimal dose for growth stimulation in tilapia. Overall, tilapias fed with FHE-coated feed exhibited significantly higher protein and energy retentions, with those fed with treatment C showed highest energy retention percentage (Table 3). However, fat retention percentage remained the same across all treatments.

## DISCUSSION

Growth occurs when the nutrients obtained from food exceed the needs for basal energy and activity (Cho & Bureau 1995). The body’s utilisation of feed is influenced by the quality of nutrients, enzymes and hormones (Bertucci *et al*. 2019). However, consuming feed in high quantity is not always beneficial, especially when the nutrients cannot be absorbed and fully utilised. In contrast, in addition to the extra cost, unnecessary excess feed will only lead to the deterioration of water quality via the increased amount of faecal matter. In this study, we found that FHE as feed additive exert positive effects on the growth and nutritional efficiency of tilapia. There was significant improvement in the growth and efficiency of tilapia treated with various doses of FHE compared to control, but with no significant detrimental effect, as shown by the high survival rate across all treatments. The optimum inclusion level for FHE in this study is within 100 mg/kg–300 mg/kg of FHE-coated feed for tilapias.

Feed utilisation efficiency is often characterised by the changes in intestinal morphology of fish. In addition, plants may contain anti-nutritional ingredients including protease inhibitors, saponins, tannins and non-starch polysaccharides that could negatively affect the normal intestinal biology of fish (Francis *et al*. 2001). For example, the use of soybean meal has resulted in reversible morphological and functional intestinal changes, such as the widening of the lamina propria, enteritis and reduction of villi and microvilli of various fish species (Sohrabnezhad *et al*. 2017; Wang *et al*. 2017; Booman *et al*. 2018). In *O. niloticus*, the use of soybean meal diets resulted in the reduction in both the height of villi and numbers of goblet cells (Obirikorang *et al*. 2020). However, the active compounds from FHE did not inhibit, but rather they act as stimulators of intestine health and growth, as exemplified in the longer and denser villi of *O. niloticus* after fed with FHE-coated diets. Intestinal villi play key roles in digestion, absorption, secretion and immunity functions. Also, the length and width of the intestinal villi are positively correlated with their nutrient absorption ability (Poolsawat *et al*. 2020). Thus, enhancement of villi length and density of *O. niloticus* after subjected to FHE-coated feed indicates that FHE promote better nutrient absorption ability by increasing larger nutrient absorptive surface in fish.

Increased immune response was also implicated from increase in the number of goblet cells and lymphocytes found in the intestinal villi of *O. niloticus* fed with FHE-coated diets. The goblet cells in fish are under direct regulation by the immune system (Birchenough *et al*. 2015). These mucus-secreting cells play a role in facilitating the excretion of digesta, mucus and mucus associated anti-microbial substances along the enterocyte space of the small intestine (Sklan *et al*. 2004; Bosi *et al*. 2017). The intestinal lymphocytes maintain gut integrity and immune homeostasis (Ma *et al*. 2019). A decrease in lymphocyte counts is often observed when fish is subjected to stress, diseased or intoxicated by heavy metals (Guo & Dixon 2021). The significant improvement of goblet cell and lymphocyte counts observed in this study highlights the potential immune enhancement of FHE-coated diet on *O. niloticus*. In addition, the potential improvement in immune response of *O. niloticus* could be due to the curcumin content found in the FHE. Similar study by Ashry *et al*. (2021) reported that the addition of curcumin to tilapia feed resulted in an increased phagocytic activity, and a reduction in the total number of bacteria. Eng-Chong *et al*. (2012) reported that the active compound of *B. rotunda*, one of the main ingredients of FHE, inhibits biofilm formation by fish intestinal pathogens. However, further study involving stress response tests is warranted to validate the positive impact of FHE-coated feed and the involvement of curcumin on the immune response enhancement of *O. niloticus*.

Improved feed utilisation was reflected in the increased nutrient and energy retention of *O. niloticus*. Elevated protein retention increases the availability of raw materials to synthesise enzymes and hormones that are important for anabolic reactions, subsequently promote growth. Furthermore, the energy to support anabolic reactions, osmoregulation and adaptation (Hvas *et al*. 2018), as represented by energy retention, was high in fishes that received FHE. The higher protein retention percentage observed in tilapias fed with FHE-coated feed could be attributed to the presence of mulberry leaf extract, a known source of phytoecdysteroids (Fujaya *et al*. 2018). Phytoecdysteroids increase protein synthesis of vertebrates by elevating intracellular calcium and sustained activation of Akt pathway (Gorelick-Feldman *et al*. 2010). Besides containing phytoecdysteroids, the overall FHE (Table 1) also contains important nutrients that are involved in the acceleration of the protein synthesis process, including iron, riboflavin, vitamin C, vitamin K, potassium, phosphorus and calcium (Das *et al*. 2021). In addition, the presence of significant amount of vitamin C (406.854 μg of vitamin C per mL of FHE) in the FHE could also contributed to the positive results obtained in this study. Fish is unable to *de novo* synthesise vitamin C and must obtain it from exogenous source (Fracalossi *et al*. 2001). Sufficient level of vitamin C resulted in enhanced growth rate and antioxidant activity, elevated growth hormone level, improved survival against *Aeromonas sabria*, and improved intestinal histomorphology (e.g., increase villi height and width) of *O. niloticus* (Ibrahim *et al*. 2020).

The fermentation process carried out on the herbal extracts used in this study had a positive effect on tilapia fingerlings. Fermentation is a powerful method that improves the total and extractable major and trace minerals of corn genotypes, while reducing phytate; a common compound that act as cation storage in plants (Sokrab *et al*. 2014). The presence of phytate adversely affect the overall growth, utilisation of nutrient and energy, and uptake of mineral in fish (Kumar *et al*. 2012). Also, the enhanced microbial activity during fermentation improves the bioavailability of phytochemicals, and macro- and micronutrients, in addition to removing anti-nutritional factors (Samtiya *et al*. 2021). Therefore, future research on the characterisation of the nutrient, biochemical and microbial community changes within FHE during the fermentation process is warranted.

This study shows that FHE is a promising functional feed additive in freshwater fish farming. The decline in growth performance of tilapia fingerlings at the highest (500 mg/kg of feed) FHE incorporation concentration might implicate that the optimum inclusion level has been exceeded. Herbal compounds/ingredients, when used excessively exceeding the recommended dose, could cause unfavourable adverse effects (Kaur *et al*. 2013; Zhang *et al*. 2015). Based on the results of this study, an optimal dose of 300 mg/kg of FHE-coated feed is recommended for the culture of tilapia fingerlings. Improving growth and feed efficiency is critical as it translates to reducing cultivation time and costs. FHE produced from local herbs certainly has its advantages in terms of supply and price of raw materials. The future application of FHE in other growth stages of tilapia and other fish and shellfish species would be feasible as it can be easily coated onto pellets of varying sizes.

## Figures and Tables

**Table 1:** The proximate composition (%) of feed used.

Proximate composition	Percentage (%)
Moisture (%)	6.83
Crude protein (% dry weight)	22.45
Crude lipid (% dry weight)	8.18
Ash (% dry weight)	8.81
Crude fibre (% dry weight)	7.76
NFE (nitrogen free extract) (% dry weight)	52.80

**Table 2:** Ingredient of the FHE used in this study.

Ingredient	Weight
Phytochemical^1^	μg/mL

Sitosterol	5.50
Alkaloid	56.17
Saponin	0.69
Flavonoid	543.65
Curcumin	0.10

Vitamin^2^	μg/mL

Vitamin A	0.18
Vitamin C	406.85

Mineral^2^	μg/mL

Phosphorus (P)	<0.05
Iron (Fe)	11.62
Potassium (K)	1536.81
Calcium (Ca)	172.17
Magnesium (Mg)	219.66
Natrium (Na)	729.31
Zink (Zn)	0.55

Enzymes^3^	U/mL

Protease	0.272
Amylase	0.122
Lipase	0.875

*Notes:*

1 Integrated research and testing laboratory of Gadjah Mada University, Indonesia,

2 Makassar Health Laboratory Centre, Indonesia, and

3 Biochemical Laboratory of FMIPA-UNHAS, Indonesia; 100 mL of FHE is equivalent to 500 mg of herbal extract (HE).

**Table 3:** Growth performances, survival rate, feed efficiency, intestinal changes and protein, fat and energy retentions of tilapia fed with diets coated with different concentrations of FHE (*n* = 3).

Parameter	Treatment			

	A	B	C	D
WG (g)	9.52 ± 1.73^a^	10.72 ± 0.63^b^	12.48 ± 0.89^b^	11.24 ± 1.08^ab^
ADG (g/day)	0.27 ± 0.05^a^	0.31 ± 0.02^a^	0.36 ± 0.02^ab^	0.32 ± 0.03^a^
SGR (%/day)	6.41 ± 0.51^a^	6.78 ± 0.17^ab^	7.21 ± 0.21^b^	6.90 ± 0.28^ab^
SR (%)	86.67 ± 7.19^a^	95.24 ± 4.36^a^	91.43 ± 7.56^a^	86.67 ± 7.19^a^
TWG (g)	237.51 ± 78.27^a^	339.89 ± 14.70^b^	360.39 ± 62.62^b^	291.25 ± 21.95^ab^
DC (g)	30.42 ± 0.98^a^	30.56 ± 2.19^a^	30.37 ± 0.54^a^	29.87 ± 1.92^a^
FE (%)	21.19 ± 6.90^a^	31.83 ± 1.23^b^	33.84 ± 5.33^b^	28.95 ± 1.27^ab^
VL (μm)	119.1 ± 4.10^a^	157.18 ± 7.87^b^	183.96 ± 7.00^c^	114.49 ± 8.59^a^
DV (μm)	31.40 ± 2.64^a^	23.46 ± 2.64^b^	19.00 ± 1.59^c^	28.98 ± 2.10^a^
GC (Cells/villi)	132.50 ± 2.12^a^	176.33 ± 8.50^b^	162.33 ± 9.02^c^	137 ± 4.58^a^
LC (cells/villi)	436 ± 18.38^a^	525.33 ± 10.26^c^	476 ± 11.79^b^	462.33 ± 5.03^b^
Protein retention (%)	46.37 ± 10.71^a^	61.71 ± 3.72^b^	68.64 ± 7.65^b^	64.95 ± 5.48^b^
Fat retention (%)	37.3 ± 4.43^a^	29.12 ± 1.90^a^	48.86 ± 3.12^a^	39.42 ± 3.13^a^
Energy retention (%)	19.66 ± 4.38^a^	22.20 ± 1.32^ab^	29.11 ± 3.13^c^	25.21 ± 2.13^bc^

*Note*: Different letters in the column are statistically different according to the LSD test (*P* < 0.05). Treatment (T): (A) 0 mg/kg of feed, (B) 100 mg/kg of feed, (C) 300 mg/kg of feed, (D) 500 mg/kg of feed; Weight Gain (WG); Average Daily Growth (ADG); Specific Growth Rate (SGR); Survival Rate (SR); Total Weight Gain (TWG); Daily Consumption (DC); Feed Efficiency (FE); Villi Length (VL); Distance between villi (DV); Goblet Cells count (GC); Lymphocyte Cell count (LC).

## Data Availability

The datasets generated during and/or analysed during the current study are available from the corresponding author on reasonable request.

## APPENDIX

Treatment:

A (0 mg/kg of feed);
B (100 mg/kg of feed);
C (300 mg/kg of feed);
D (500 mg/kg of feed)

Proximate analysis[Table t4-tlsr-34-2-39]

**Table t4-tlsr-34-2-39:**

Treatment	Moisture (%)	(% dry weight)				

		Crude protein	Crude lipid	Ash	Crude fiber	Nitrogen free extract (NFE)
Feed	6.83	22.45	8.18	8.81	7.76	52.8
Initial Fish	75.72	61.19	3.48	11.02	0.79	23.52
After treatment A	74.92	73.49	12.88	8.37	0.77	4.49
After treatment B	77.03	76.78	9.32	8.08	0.82	5
After treatment C	75.03	72.47	12.85	9.15	0.67	4.86
After treatment D	74.04	74.31	11.2	7.87	0.79	5.83
